# The longitudinal impact of low-dose morphine on diurnal cortisol profiles in people with chronic breathlessness and chronic obstructive pulmonary disease (COPD): an exploratory study

**DOI:** 10.1186/s12931-025-03230-9

**Published:** 2025-04-23

**Authors:** Diana H. Ferreira, Richella Ryan, Nina Smyth, Angela Clow, David C. Currow

**Affiliations:** 1 Graduate School of Medicine, Faculty of Science, Medicine and Health, University of Wollongong, Wollongong, NSW Australia; 2 Arthur Rank Hospice Charity, Cherry Hinton Road, Shelford Bottom, CB22 3FB Cambridge, UK; 3 University of Westminster, Regent St, W1B 2HW London, UK; 4 Faculty of Health, University of Technology Sydney, Sydney, NSW 2007 Australia

**Keywords:** Chronic breathlessness, Sustained-release morphine, Cortisol, Palliative care, Symptom control

## Abstract

**Introduction:**

Stress activates the hypothalamic-pituitary-adrenal (HPA) axis of which cortisol is an end product. ‘Allostatic load’ is where systems including the HPA axis are exposed to high, cumulative, physiologic burdens (such as chronic breathlessness) leading to flatter diurnal cortisol slopes and poorer health outcomes. The aim of this hypothesis-generating study explored longitudinal changes in cortisol secretion and any associated changes in breathlessness after introducing regular, low dose morphine or placebo.

**Methods:**

This was an optional, hypothesis-generating sub-study embedded in a multi-site, randomised, double-blind, placebo-controlled trial (RCT) of regular, low-dose morphine for chronic breathlessness and chronic obstructive pulmonary disease. In a blinded dose-increment algorithm by week three, doses were 0 mg-32 mg. Participants in the RCT could elect to continue in a six-month blinded extension. This sub-study excluded people who used non-inhaled corticosteroids in the previous month or were on subcutaneous insulin. Participants collected saliva for cortisol assays for two days at baseline, and ends of weeks 1, 3 and 12 at 3,6 and 12 h after waking, generating sufficient data to calculate diurnal cortisol slopes and areas under the curve (AUC). Samples were analysed using ELISA. Correlations between diurnal cortisol profiles (slope and AUC) and a range of measures were explored.

**Results:**

Twenty mostly female former smokers were in this sub-study. At baseline and the end of week 1, one-way ANOVA between-group analyses showed no significant differences in the log-transformed cortisol slope or ln-AUC. There was a strong correlation between the age-adjusted Charlson Comorbidity Index (CCI) and ln-AUC (*r*=-0.70, *p* < 0.001) and moderate correlation with age (*r*=-0.43, *p* = 0.06). In the blinded extension study, there was a self-selecting blinded group (*n* = 7) all on active medication. Global impression of change (GIC) was highly correlated with the diurnal cortisol slope (rs = 0.98, *p* = 0.01), and with decrease in *average breathlessness* (*r* = 0.89, *p* = 0.04).

**Discussion:**

This hypothesis-generating study did not show a relationship between the diurnal cortisol profile and morphine in people with chronic breathlessness and COPD. For the sub-group still on study at 12weeks, the cortisol curves became steeper as *average breathlessness* decreased and as global impression of change (GIC) improved, suggesting that reducing breathlessness may potentially positively impact the HPA axis in a sub-group of people.

**Trial registration:**

Registration Number NCT02720822 date registered 28/03/2016.

**Supplementary Information:**

The online version contains supplementary material available at 10.1186/s12931-025-03230-9.

## Introduction

Stress is defined as “a state of real or perceived threat to homeostasis that may challenge an organism’s well-being” [1]. The *stress response* is characterised by a complex chain of physiologic and behavioural changes supporting the return to homeostasis [2–4]. This includes activation of the hypothalamic-pituitary-adrenal (HPA) axis which plays a key role in sustaining, modulating and terminating the stress response [1]. Cortisol is an end product of the HPA axis, which is released by the adrenal cortex and responsible for promoting a range of metabolic, cardiovascular, behavioural and immune adaptive responses [5, 6]. Cortisol release is regulated by a negative feedback loop to the hypothalamus and pituitary.

‘Allostatic load’ is where allostatic systems such as the HPA axis are exposed to sufficiently high, cumulative, physiologic burden that they become disrupted and damage the body [7, 8]. For example, excess production of inflammatory cytokines or chronic stress may disrupt the HPA axis, cardiovascular, and central nervous systems, forcing each system to compensate [9, 10]. Higher allostatic loads, identified by at least three different biological markers, were shown to be associated with worse physical and mental health across different settings [8, 11, 12]. Allostatic load on the HPA axis is classically identified by disruption to the normal patterns of diurnal cortisol secretion [13] and are indicative of disrupted circadian signalling [14]). In particular, flatter diurnal cortisol slopes are associated with a wide range of poorer emotional and physical health [15].

Cortisol can be accurately, conveniently and non-invasively measured in saliva, even in people with a dry mouth [16, 17]. In saliva, cortisol is present in its free form and is therefore less prone to fluctuations due to changes in erythrocytes or serum protein– changes that may be present in people with chronic breathlessness.

Changes in the cortisol circadian rhythm may be particularly relevant in chronic unremitting conditions [18]. Importantly, the diurnal cortisol secretion rhythm seems to involve two different patterns [19]: the post-awakening rise in cortisol levels and a basal diurnal cortisol curve reflecting changes from mid-morning to evening. These responses need to be evaluated separately [19].

Chronic breathlessness may be associated with stress and higher allostatic load because it is:

1. a potentially life-threatening sensation;
2. a source of fear and anxiety; and.
3. frequently prolonged and progressive, leading high to physiologic burden.

Additionally, people with chronic breathlessness are typically older and have multiple comorbid conditions generating multiple symptoms, contributing to even higher allostatic loads [20, 21]. Theoretically, higher allostatic loads may be one of the mechanisms by which chronic breathlessness contributes to premature mortality [22, 23].

A link between chronic breathlessness and HPA dysregulation was suggested by one cross-sectional study of 110 participants, comparing the salivary diurnal cortisol profile of people with chronic breathlessness with those of healthy participants [23]. This study revealed that people with moderate-to-severe *breathlessness limiting exertion* (modified Medical Research Council [mMRC] breathlessness scores of 2–4 on a five point ordinal scale) have dysregulation of their normal circadian rhythm of cortisol production generating flatter diurnal cortisol slopes compared with people with mild or no *breathlessness limiting exertion* (mMRC 0 or 1), or healthy controls (*p* < 0.001 in the ANCOVA model for both comparisons).

There is a need to understand better the patterns of cortisol secretion in people with chronic breathlessness. Understanding these patterns is critical to identifying factors that may generate or sustain the observed poorer wellbeing and long-term health outcomes for people who report chronic breathlessness. Further, if the intensity in breathlessness can be reduced, could this be reflected in improved physiological parameters such as restoration of a more normal pattern of diurnal cortisol secretion reflecting a physiological response to better symptom control. Regular, low dose, sustained-release morphine is a potential therapy to reduce the symptoms of chronic breathlessness which requires evaluation [24] of any effect on the HPA axis function.

The aim of this hypothesis-generating study was to explore:

1. patterns of cortisol secretion in people with chronic breathlessness (extending the earlier cross-sectional findings); and.
2. any changes in patterns of cortisol secretion associated with changes in breathlessness intensity after the introduction of regular, low dose, sustained-release morphine (by dose) or placebo for chronic breathlessness.

## Methods

This prospective study was an optional, hypothesis-generating sub-study embedded in a multi-site, randomised, double-blind, placebo-controlled trial (RCT) of regular, low-dose, sustained-release morphine for chronic breathlessness associated with chronic obstructive pulmonary disease (COPD). Given block randomisation, there was a representative sample of the varying doses of sustained-release morphine to which people were exposed.

**Fig. 1:**
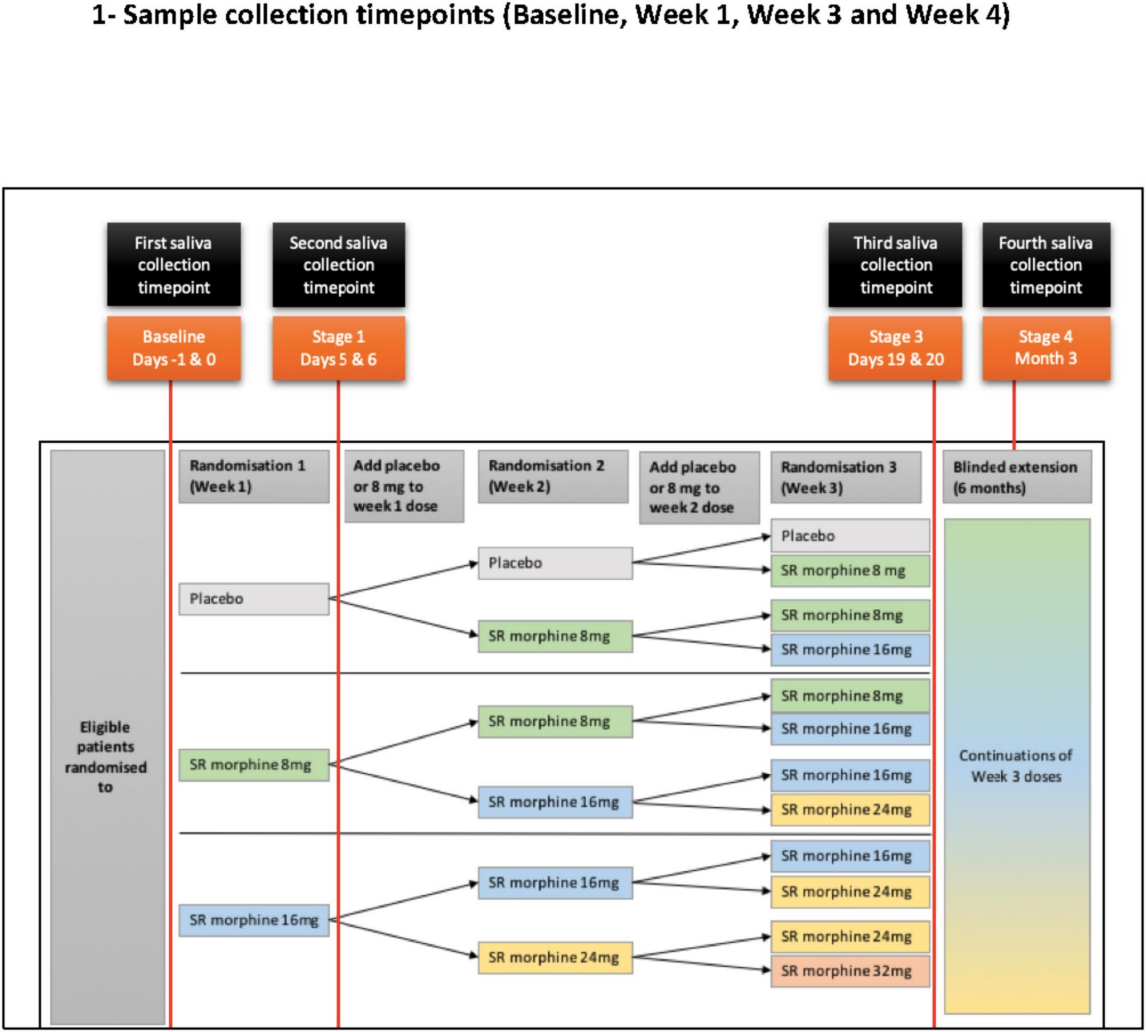
Figure 1

Design and conduct of the RCT are described in detail elsewhere [25]. In brief, participants were randomised to one of three arms: once-daily placebo, sustained-release morphine 8 mg or 16 mg for one week. Irrespective of the symptomatic response, they were further randomised at weeks 2 and 3 to add placebo or 8 mg of sustained release morphine to their initial dose. At week three, doses ranged from 0 mg to 32 mg of sustained release morphine daily. An optional six-month blinded extension was available to all participants. (Fig. 1)

In addition to the eligibility criteria for the main study (Web appendix 1), this study required exclusion of people who had oral or intravenous corticosteroids in the previous four weeks (given their disruption of the HPA axis *and* cross-reactivity with salivary cortisol immunoassays; [26]) or were on subcutaneous insulin for diabetes (given described changes in the cortisol awakening response and the diurnal cortisol slope [27, 28]).

Participants were asked to collect saliva samples at four timepoints across the whole study (Baseline (day − 1, 0), end of week 1 (days 5 and 6; steady state for week 1 dose [29]), end of week 3 (days 19 and 20; steady state for week 3 dose), and blinded extension (after taking stable (blinded) medication for at least three months; Fig. 1). For each timepoint, participants were provided with a saliva self-collection pack, a diary for collection days, and an instruction sheet. Each saliva self-collection pack included six saliva-collection devices to collect three daily saliva samples (3, 6 and 12 h after waking) across two consecutive days generating sufficient data to calculate the diurnal cortisol slope and AUC for each participant at each of the collection timepoints [30, 31]. Participants were asked to take steps to minimise stress, especially in the thirty minutes before each sample’s collection.

Saliva samples were collected using the Salivette^®^ Cortisol devices (SARSTEDT, Australia), each consisting of a synthetic swab stored in a plastic tube, together with contemporaneous diary data. Participants were instructed to chew on the swab for one minute before re-storing it in the pre-labelled, colour-coded plastic tube, according to the manufacturers’ instructions. Salivary cortisol is relatively stable at room temperature and did not require freezing but samples were stored in people’s refrigerators until collection by study staff (up to one week later). Samples were frozen at -30^0^ in a central repository until the completion of the whole study and then transported on dry ice to the Stratech^R^ facility in NSW for analysis in September, 2019. All samples underwent one freeze-thaw cycle. On the day of the assay, all samples were thawed and analysed using an Enzyme Linked Immunosorbent Assay (ELISA) developed by Salimetrics LLC (USA), according to the manufacturers’ instructions. Performance testing by Salimetrics indicates a high correlation between salivary cortisol and matched serum cortisol concentrations (*r* = 0.91). The lower limit of detection (assay sensitivity) is 0.08 nmol/L.

### Sample size and statistical analyses

Considering that this was a hypothesis-generating study embedded in a larger RCT, no formal power calculation was undertaken. Changes in diurnal cortisol slope and AUC were examined while accounting for other variables which could potentially induce HPA changes, including:

- breathlessness scores measured with a numerical rating scale (NRS) for *worst*, *average*, and *unpleasantness* of breathlessness in the previous 24 h; higher scores indicate more intense breathlessness [32, 33];
- modified Medical Research Council (mMRC) breathlessness scale scores; higher scores indicate greater limitations to exertion because of breathlessness [34, 35];
- performance status assessed with the Australia-modified Karnofsky Performance Scale (AKPS); lower scores reflect poorer function [36];
- quality of life measured using the EuroQual (EQ-5D-5 L) tool [37];
- subjective sleep quality measured with a 4-point Likert scale - from 1 (very good) to 4 (no sleep at all) [38];
- anxiety and depression scores measured with the Hospital Anxiety and Depression Scale (HADS); higher scores indicate the greater likelihood of anxiety and/or depression [39, 40];
- global impression of change (GIC) with the study medication (i.e., regular, low-dose, sustained-release oral morphine or placebo) measured with a 7-point Likert scale; from *much worse *through a neutral perception to *much better* [41]; and.
- harms commonly associated with morphine (constipation, nausea, drowsiness) measured with 4-point Likert scales (higher scores indicate greater harms).

Normality tests (Shapiro-Wilk) were conducted for all numerical variables to determine the appropriate statistical tests. Raw salivary cortisol concentrations were highly skewed and therefore logarithmic transformation (Ln) was employed to normalise data distribution and facilitate inferential statistics [42, 43]. The impact of non-compliance on diurnal cortisol slopes and AUC was assessed by comparing means of compliant and non-compliant groups at each sub-study stage using unpaired t-tests (with participants with unrecorded collection times included in the non-compliant group).

For Baseline and Week 1, between-arm comparisons were conducted using one-way ANOVA. As the number of participants at Weeks 3 and 4 were smaller, statistical between-arm comparisons were not performed and these data are only described.

Relationships between diurnal cortisol profile (slope and AUCg) and breathlessness measures, other self-assessment measures and harms were explored at each week using Pearson’s or Spearman’s correlation depending on the nature of the variable examined (i.e., numeric or ordinal) and its distribution (i.e., presence or absence of normal distribution). Potential influences of participants’ baseline characteristics on the diurnal cortisol profile (slope and AUCg) were explored using Pearson’s correlation for numerical variables, and unpaired t-tests for comparison of sub-groups defined by categorical variables.

### Ethics approval / additional written informed consent

This study was approved as a sub-study of the BEAMS trial [25] by the Hunter New England Human Research Ethics Committee (Reference 15/12/16/3.06) and by the local Research Governance Office at each site in accordance with the Declaration of Helsinki. The BEAMS Trial is registered with the National Institutes of Health Clinical Trials Registration site (NCT02720822). In addition to written, informed consent for the main RCT, participants in this sub-study provided additional written, informed consent.

### Reporting framework and data

This sub-study is reported using the CONSORT framework for reporting randomised controlled studies. No data were imputed.

## Results

The main BEAMS study did not show any differences in the reduction of breathlessness between morphine and placebo [25], so differences in breathlessness between groups were not expected in this sub-study. The study explores any impact of regular, low-dose, sustained release morphine on the HPA axis independent of any effects on breathlessness.

### Study sample

From the 156 participants included in the BEAMS study, 105 were screened for this sub-study between August 2017 and August 2019: 27 were excluded because they were taking or had taken systemic glucocorticoids in the previous four weeks; 2 were taking insulin for diabetes and 53 chose not to participate. Twenty-three participants were eligible and were included in the cortisol sub-study. Of these people, three were excluded from the analysis for: skipping baseline collections by mistake; collecting invalid baseline samples; and lack of compliance by omission of multiple collection times.

Participants were mostly elderly women who were overweight, able to care for themselves but unable to carry on normal activity or do active work (AKPS ≤ 70; Table 1). Most participants were former smokers and only two participants required long-term oxygen therapy. Approximately one half of the participants had current histories of psychiatric illnesses (e.g., anxiety and/or depression), and more than one half of those people were taking long-term antidepressants. Most participants were medicated with a steroid inhaler, whose doses had been stable for ≥ 1 week prior to sub-study enrolment.

**Table 1:** Baseline characteristics of the 20 participants of the cortisol sub-study (clinician assessment measures), separated by study arm according to their allocation at baseline

Characteristics		Placebo (*n* = 6) mean ± SD median (IQR)	SR morphine 8 mg (*n* = 8) mean ± SD median (IQR)	SR morphine 16 mg (*n* = 6) mean ± SD median (IQR)	Differences between arms*
**Age (years)**		71.8 ± 2.9 72.0 (70.0–74.3)	68.3 ± 8.3 69.0 (62.0–71.0)	72.0 ± 12.4 74.0 (65.5–80.5)	H(2) = 1.69, *p* = 0.26
**Sex (male)**		5 (83.3%)	3 (37.5%)	1 (16.7%)	χ^2^ = 5.69, *p* = 0.06
**BMI (kg/m** ^**2**^ **)**		30.0 ± 5.3 30.9 (25.2–34.5)	31.9 ± 7.5 33.0 (29.0–35.4)	24.8 ± 8.2 22.7 (17.4–33.1)	F = 1.66, *p* = 0.22
**Charlson Comorbidity Index - age adjusted**		5.2 ± 1.2 4.8 (4.0–6.3)	4.8 ± 1.5 5.0 (4.0–6.0)	5.3 ± 1.0 6.0 (4.0–6.0)	H(2) = 0.64, *p* = 0.73
**AKPS**	**80**	-	2 (25.0%)	2 (33.3%)	H(2) = 1.40, *p* = 0.50
	**70**	4 (66.7%)	3 (37.5%)	3 (50.0%)	
	**60**	2 (33.3%)	3 (37.5%)	1 (16.7%)	
**Smoking status**	**Current smoker**	-	2 (25.0%)	1 (16.7%)	χ^2^ = 3.56, *p* = 0.47
	**Former smoker**	6 (100%)	5 (62.5%)	5 (83.3%)	
	**Never smoked**	-	1 (12.5%)	-	
**End tidal CO**_**2**_ **(mmHg)** ^**a**^		30.0 ± 7.3 32.0 (21.8–35.3)	29.4 ± 4.6 28.5 (26.1–34.0)	24.8 ± 4.8 26.3 (21.3–28.3)	F = 1.63, *p* = 0.23
**Supplemental oxygen**		1 (16.7%)	1 (12.5%)	1 (16.7%)	χ^2^ = 2.84, *p* = 0.58
**Relevant health conditions**	Diabetes mellitus (type I or II)	0 (0.0%)	1 (12.5%)	1 (16.7%)	χ^2^ = 1.02, *p* = 0.60
	Current history of psychiatric illness	3 (50%)	4 (50%)	2 (33.3%)	χ^2^ = 0.47, *p* = 0.79
	Previous history of psychiatric illness	0 (0%)	1 (12.5%)	0 (0%)	χ^2^ = 1.93, *p* = 0.38
**Relevant Medication**	Steroid inhaler	4 (66.7%)	7 (87.5%)	7 (100%)	χ^2^ = 2.68, *p* = 0.26
	Anti-depressant	3 (50.0%)	2 (25.0%)	1 (16.7%)	χ^2^ = 1.75, *p* = 0.42
	Hormone therapy	0 (0%)	1 (12.5%)	0 (0%)	χ^2^ = 1.58, *p* = 0.45
	Immunosuppressant	0 (0%)	2 (25%)	1 (16.7%)	χ^2^ = 1.70, *p* = 0.43
	Chemo- or radiotherapy	0 (0%)	0 (0%)	0 (0%)	-
**Availability of a carer**		4 (66.7%)	6 (75.0%)	5 (83.3%)	χ^2^ = 0.07, *p* = 0.97
**mMRC**	Grade 3	5 (83.3%)	8 (100%)	6 (100%)	χ^2^ = 2.46, *p* = 0.29
	Grade 4	1 (16.7%)	-	-	
**Breathlessness**	*Worst*	6.3 ± 2.2 6.0 (4.6–8.6)	4.5 ± 1.5 4.8 (3.0–5.9)	5.9 ± 1.5 6.3 (4.6–6.9)	F = 2.18, *p* = 0.14
	*Average*	5.1 ± 2.3 4.0 (3.4–7.8)	3.7 ± 1.1 3.8 (2.6–4.9)	4.7 ± 1.4 4.8 (3.4–5.5)	H(2) = 1.67, *p* = 0.43
	*Distress caused by breathlessness*	3.8 ± 3.4 2.3 (1.3–7.5)	2.8 ± 2.4 2.3 (0.5–4.8)	4.3 ± 2.9 4.8 (1.3–6.3)	F = 0.45, *p* = 0.64
**Quality of life (EQ-5D-5 L**,** VAS)**		65.7 ± 23.9 60.0 (52.5–88.5)	51.3 ± 18.9 52.5 (32.5–71.3)	62.5 ± 24.8 62.5 (37.5–90.0)	F = 0.83; *p* = 0.45
**Hospital Anxiety and Depression Scale (HADS)**	HADS (Anxiety, 0–21)	7.0 ± 4.9 6.0 (3.8–12.3)	7.3 ± 4.2 8.0 (4.0–11.0)	4.8 ± 3.8 5.0 (1.5–7.8)	F = 0.61; *p* = 0.56
	HADS (Depression, 0–21)	6.2 ± 4.1 5.5 (3.3–9.3)	6.3 ± 3.9 5.0 (3.0–9.0)	4.5 ± 3.8 3.0 (1.8–8.0)	F = 0.41; *p* = 0.67
**Quality of sleep**	Very good	1 (16.7%)	1 (12.5%)	1 (16.7%)	χ^2^ = 7.95, *p* = 0.24
	Good	4 (66.7%)	3 (37.5%)	1 (16.7%)	
	Poor	-	4 (50%)	4 (66.7%)	
	No sleep at all	1 (16.7%)	-	-	

BMI = body mass index, VAS = Visual Analogue Scale. Symbols: *Statistical tests performed in order of reporting: Kruskal-Wallis test (non-normally distributed numerical variables), Chi-squared test (categorical variables), and one-way ANOVA (normally distributed numerical variables),

^*a*^ *Measured with a portable capnography device during quiet breathing.*

### Compliance with cortisol sampling

Eighty-three (83/120; 69.2%) baseline collections (20 participants times six samples) were collected within one hour of the recommended collection times (Table 2).

**Table 2:** Compliance with collection times by study stage

		n (expected number of samples = n x 6)	Collection compliance and quality n (row %)			
			Within 1 h of designated time	Within 3 h of designated time	No time recorded**	Missing sample
Study stage	Baseline	20 (120)*	83 (69.2)	4 (3.3)	33 (27.5)	-
	**Stage 1** End of week 1	**17 (102)**	**55 (53.9)**	**3 (2.9)**	**39 (38.2)**	**5 (4.9)**
	**Stage 3** End of week 3	**11 (66)**	**63 (95.5)**	**3 (4.5)**	**-**	**-**
	**Stage 4** End of 3 months	**7 (42)**	**35 (83.3)**	**1 (2.4)**	**6 (14.3)**	**-**

*placebo *n* = 6; sustained release morphine 8 mg *n* = 8; sustained release morphine 16 mg *n* = 6

** There was no statistical difference between compliant and non-compliant sub-groups at any study stage for diurnal cortisol slope nor AUCg

### Relationship between cortisol profile and morphine

At baseline, the one-way ANOVA between-group analyses showed no significant differences between arms in the Ln-transformed summary measures of diurnal cortisol slope [F (0.24), *p* = 0.79] and AUCg [F (0.06), *p* = 0.94] (Table 3).

**Table 3:** Baseline cortisol mean/median values in each arm-

	Placebo mean ± SD median (IQR)* (*n* = 6)	SR Morphine 8 mg mean ± SD median (IQR)* (*n* = 8)	SR Morphine 16 mg mean ± SD median (IQR)* (*n* = 6)	Between-arm differences^†^
3-hour cortisol (nmol/L)	4.99 ± 4.7 4.73 (2.7–7.4)	8.69 ± 5.6 7.93 (4.3–13.3)	6.10 ± 3.3 5.08 (3.4–9.5)	-
6-hour cortisol (nmol/L)	5.69 ± 4.5 4.23 (2.2–8.9)	4.01 ± 2.1 3.77 (2.3–5.7)	4.62 ± 1.8 4.13 (3.09–6.3)	-
12-hour cortisol (nmol/L)	2.28 ± 1.2 2.04 (1.1–3.5)	2.90 ± 2.1 2.04 (1.6–3.9)	2.53 ± 1.34 2.23 (1.9–2.4)	-
Ln diurnal slope^†^ (Ln nmol/L/hr)	-0.09 ± 0.07	-0.12 ± 0.09	-0.10 ± 0.07	F = 0.24, *p* = 0.79
Cortisol AUCg (nmol.hr/L)	39.9 ± 23.7 29.9 (24.9–64.6)	39.8 ± 13.4 42.9 (28.8–49.3)	37.5 ± 12.6 31.5 (29.1–51.5)	-
Ln cortisol AUCg^†^ (Ln nmol.hr/L)	3.51 ± 0.62	3.59 ± 0.40	3.57 ± 0.32	F = 0.06, *p* = 0.94

*Values are mean ± standard deviation (SD) and median (interquartile range), except for variables with normal distribution for which values are presented as mean ± SD only

†Analysis conducted only in Ln-transformed cortisol measures with normal distribution (Levene’s test *p* ≥ 0.05)

In Week 1, there were no significant differences between the three arms (placebo, 8 mg morphine daily, 16 mg morphine daily) for log-transformed cortisol slope nor for area under the curve (Table 4). There were no correlations for clinically relevant relationships between the diurnal cortisol slope (Table 5).

**Table 4:** End of 1 week of therapy: cortisol mean/median values in each arm

	Placebo mean ± SD median (IQR) (*n* = 6)	SR Morphine 8 mg mean ± SD median (IQR) (*n* = 8)	SR Morphine 16 mg mean ± SD median (IQR) (*n* = 6)	Between-arm differences^†^
3-hour cortisol (nmol/L)	10.35 ± 6.5 9.96 (5.2–13.8)	6.46 ± 4.2 4.87 (3.7–7.8)	5.52 ± 2.0 6.18 (3.0–7.1)	-
6-hour cortisol (nmol/L)	5.78 ± 4.1 4.18 (3.8–6.9)	3.40 ± 1.8 3.02 (1.9–4.5)	3.67 ± 1.5 3.44 (2.3–4.9)	-
12-hour cortisol (nmol/L)	3.26 ± 2.1 2.45 (1.9–4.7)	2.49 ± 1.5 2.01 (1.5–3.0)	2.90 ± 1.6 2.54 (1.5–4.3)	-
Diurnal slope (Ln nmol/L/hr)	-0.12 ± 0.10	-0.10 ± 0.05	-0.08 ± 0.06	F = 0.35, *p* = 0.71
Cortisol AUCg (nmol.hr/L)	51.3 ± 22.9 44.0 (34.5–75.3)	34.8 ± 16.4 28.5 (25.0–48.6)	34.4 ± 8.3 35.2 (26.1–41.6)	-
Ln cortisol AUCg (Ln nmol.hr/L)	3.85 ± 0.39	3.44 ± 0.45	3.49 ± 0.28	F = 1.56, *p* = 0.24

*Values are mean ± standard deviation (SD) and median (interquartile range), except for variables with normal distribution for which values are presented as mean ± SD only

†Analysis conducted only in Ln-transformed cortisol measures with normal distribution (Levene’s test *p* ≥ 0.05)

AUCg– area under the curve (ground)

**Table 5:** Correlations between study measures and diurnal cortisol slope

	Ln- Diurnal slope			
	Baseline (*n* = 20)	End of week 1 (*n* = 17)	End of week 3 (*n* = 11)	At least 3 months on the blinded extension study (*n* = 7)
***Worst*** **(NRS)**	*r* = 0.33 *p* = 0.15	*r* = 0.27 *p* = 0.29	*r* = 0.33 *p* = 0.36	*r* = 0.79 *p* = 0.11
***Average*** **(NRS)**	*rs* = 0.30 *p* = 0.21	*rs* = 0.31 *p* = 0.23	*r* = 0.08 *p* = 0.82	*r* = 0.89 *p* = 0.04
***Distress*** **(NRS)**	*r*=-0.01 *p* = 0.98	*r* = 0.02 *p* = 0.93	*r*=-0.37 *p* = 0.29	*r* = 0.10 *p* = 0.87
**mMRC**	*rs*=-0.18 *p* = 0.45	*rs*=-0.45 *p* = 0.07	*rs*=-0.56 *p* = 0.08	*-*
**AKPS**	*rs*=-0.66 *p* = 0.001	*rs*=-0.12 *p* = 0.66	*rs*=-0.24 *p* = 0.49	*rs* = 0.00 *p* = 1.00
**QOL (EQ-5D-5 L VAS)**	*r* = 0.14 *p* = 0.56	*r*=-0.14 *p* = 0.59	*-*	*rs*=-0.62 *p* = 0.27
**Subjective sleep quality**	*rs*=-0.61 *p* = 0.005	*rs*=-0.42 *p* = 0.10	*rs*=-0.29 *p* = 0.43	*rs* = 0.58 *p* = 0.31
**HADS– anxiety**	*r*=-0.15 *p* = 0.54	*r* = 0.04 *p* = 0.89	*-*	*-*
**HADS– depression**	*r* = 0.09 *p* = 0.73	*r*=-0.50 *p* = 0.04	*-*	*-*
**Global impression of change**	*-*	*rs* = 0.03 *p* = 0.91	*rs* = 0.10 *p* = 0.78	*rs* = 0.98 *p* = 0.01

NRS = Numerical rating scale; mMRC = modified Medical Research Council scale; AKPS = Australian Karnofsky Performance Status; QOL (EQ-5D-5 L) **=** Quality of life measured using the EQ-5D-5 L questionnaire; HADS = Hospital Anxiety and Depression Scale. Subjective sleep quality measured with a 4-point Likert scale; Global impression of change measured with a 7-point Likert scale. Statistically significant correlations indicated in light grey

In Week 3, Ln-transformed mean diurnal cortisol slopes were similar for all three arms: sustained-release morphine ≥ 16 mg (mean ± SD = -0.12 ± 0.06, *n* = 6); sustained-release morphine 8 mg (mean ± SD = -0.11 ± 0.09, *n* = 4); and placebo (-0.10). Ln-AUCg were of a similar order of magnitude and direction: placebo (3.50); sustained-release morphine 16 mg (mean ± SD = 3.35 ± 0.92, *n* = 6); and sustained-release morphine 8 mg (mean ± SD = 3.05 ± 0.21, *n* = 4; Table 4).

At Week 12, there is a small, self-selecting group (*n* = 7) who continued with the study because they perceived benefit from the intervention while blinded. All were on active medication. Within this group the global impression of change (GIC) and correlation with the diurnal slope was extremely high (rs = 0.98, *p* = 0.01). This is in contrast to the findings at the end of Weeks 1 and 3 where no group showed any significant difference.

In the group still on medications at week 12, there were positive correlations between diurnal cortisol slope and *average breathlessness* and *worst breathlessness*, with the slope becoming flatter when both measures were more intense (*r* = 0.89, *p* = 0.04; *r* = 0.79, *p* = 0.11, respectively).

### Relationship between cortisol profile and other clinical measures

At baseline, there were no statistically significant correlations between summary cortisol measures (slope and AUCg) and any of the breathlessness measures at Baseline (Table 5). Measures of function and symptom burden that were correlated with the diurnal cortisol slope included: AKPS with the slope flattening as functional status declined (*rs*=-0.66, *p* = 0.001); and sleep quality with the slope flattening as self-reported sleep worsened (*rs*=-0.61, *p* = 0.005). Factors with the potential to influence HPA axis activity and cortisol levels were examined to explore their influence at baseline. There was a strong correlation between the age-adjusted Charlson Comorbidity Index (CCI) and the ln-AUCg, with the ln-transformed AUCg becoming lower for higher comorbidity indices (*r*=-0.70, *p* < 0.001). There was also a moderate correlation between age and Ln-AUCg, with the ln-transformed AUCg becoming lower with more advanced age (*r*=-0.43, *p* = 0.06).

## Discussion

This hypothesis-generating study did not show a clear relationship between the diurnal cortisol profile and therapy with regular, low-dose, sustained-release morphine in the context of chronic breathlessness associated with COPD. These results are consistent with the main trial, where there were no differences in breathlessness scores between the morphine and placebo groups after one week [25]. Data at baseline provided support for links between diurnal cortisol profiles with age and number of co-morbidities. Worsening physical functioning and poorer sleep were also associated with flatter cortisol curves at baseline. For the sub-group remaining on this study for 3 months, the cortisol curves became steeper as *average breathlessness* decreased and as global impression of change (GIC) improved, suggesting that morphine may potentially have an impact on the HPA axis long term, at least in a sub-group of people.

The study builds directly on the seminal work of Ryan et al. [23]., replicating the success of monitoring HPA axis function using self-collected saliva sampling in a population of people with COPD and a range of co-morbidities. Determination of cortisol profiles requires saliva sampling according to a strict time schedule, relative to awakening time [19]. Here we chose to collect samples at 3,6 and 12 h post awakening. The majority of participants collected samples within an hour of the required time. There were no statistically significant differences in cortisol values between samples collected late or on time as all samples were collected after the initial post-awakening burst in cortisol secretion when values are critically time dependent [19]. 

The cross-sectional study of Ryan et al. [23]. demonstrated flatter mean diurnal cortisol slope in people with more intense *breathlessness limiting exertion* (MRC), but not intensity rating of breathlessness potentially reflecting that it is overall function that has the strongest association with changes in diurnal cortisol secretion patterns. The current study would support the direction of these associations at Weeks 1 and 3.

No healthy control group was studied in the current study but it is interesting to cautiously observe values between studies. Ryan et al.. reported cortisol values at 3-, 6- and 12-hours post awakening for a similar age group of healthy participants as: 15.3 ± 8.2; 8.3 ± 4.1; 2.6 ± 1.4nmol/l. Concentrations at 3 and 6 h post awakening are clearly higher in that healthy control sample compared to our study sample (and the clinical sample reported in Ryan et al..). However, the 12-hour values are similar in both studies for all participants, accounting for the reduced diurnal decline reported. A reduced diurnal decline in cortisol secretion has been associated with a wide range of physical and mental ill-health [15] and is indicative of disrupted circadian function [14]. 

In this current study, associations with diurnal cortisol secretion were most evident at baseline, where statistical power was greatest. Although no direct correlations with breathlessness measures were found, data were consistent with expectations with flattened profiles being associated with worse functional status and worse sleep quality. The negative correlation between age and the age-adjusted Charlson Comorbidity Index (CCI) with total cortisol secreted across the day reflects that healthy ageing is associated with greater cortisol secretion [44, 45]. We attribute this to circadian dysfunction in this population and the reduced diurnal slope, resulting in lower cortisol concentrations at 3 and 6 h post awakening, as discussed above.

At three months, the results of a steeper cortisol diurnal slope being associated with more positive global impression of change and less NRS-rated breathlessness are consistent with reports that sub-groups of people respond to morphine administration [24]. These responders may undergo restoration of a more dynamic and healthy pattern of diurnal cortisol secretion [15] and provide the first evidence that symptomatic relief of chronic breathlessness has benefits for broader aspects of wellbeing.

In seeking the first longitudinal pilot data, this current study adds an important dimension to the potential negative impacts of chronic breathlessness (loss of the diurnal pattern of cortisol secretion) and the theoretical benefit of working to relieve the symptom with the potential of a concomitant physiological improvement with systemic benefits. The work opens a wide-ranging line of inquiry into the negative pathophysiological impacts of chronic breathlessness and the potential of reversing some of these impacts with better symptom control (when the underlying reversible cause(s) is/are optimally treated).

A strength of this study is that it is a prospective collection of data when people were still blinded to the arm to which they were randomised allowing analyses to include the subjective sensation of breathlessness in the most objective way. The assay used is a well validated tool and participant compliance was relatively high. Sufficient sampling across 12 h was considered sufficient to calculate parameters including diurnal decline and AUCg. The study demonstrates the feasibility and need to extend this line of investigation to clearly establish treatment effects upon the HPA axis function.

Limitations include the small sample size that diminished further as time on study progressed and the absence of other supporting physiological measures that may reflect the net impact of reduced symptom burden. Further, only one person had the most severe level of breathlessness (mMRC 4) in this sub-study, with evidence from this trial and earlier studies that people with the most severe chronic breathlessness derive the most symptomatic benefits from regular, low-dose morphine [46].

The study reflects the complexity of changes in patterns of cortisol secretion in people with COPD and chronic breathlessness. It points to the need for larger, prospective studies in people who perceive that their chronic breathlessness has responded symptomatically to the therapy in order to understand more completely any physiological changes that may occur as the pharmacodynamic effects of regular, low dose sustained release morphine are realised.

### Implications for future research

This current small study is one further step towards understanding any interplay between chronic breathlessness and the HPA axis. Considering the longitudinal changes seen, this study needs to be replicated on a much larger scale in an unblinded consecutive cohort of people commenced on regular, low dose sustained release morphine for the symptomatic reduction of chronic breathlessness. In designing such a study, learning from the challenges of recruitment to the current study will be invaluable. This current study shows that such a study is necessary given that one third of participants, all of whom were on active medication, continued to take that medication at three months and a strong correlation with their diurnal cortisol secretion curves. If there are a cohort of responders who re-establish a ‘healthier’ diurnal pattern of cortisol secretion, does this benefit other measures of health and wellbeing?

### Implications for clinical care

There are no direct implications for day-to-day clinical care, however there is the possibility that, as clinicians, we have under-estimated the benefits of symptom control, dismissing it in our minds simply as a ‘nice to have’ outcome. Were there measurable systemic benefits, it fundamentally shifts the responsibility of clinicians to be much more proactive in seeking the presence and impact of symptoms, and responding in timely and fulsome ways [47, 48].

Most importantly, this work may help to characterise the sub-group of people who can safely benefit from regular, low-dose sustained release morphine for the symptomatic reduction of chronic breathlessness. Although the size of such a group may be relatively small, the benefit that they derive is potentially lifechanging [49].

## Electronic supplementary material

Below is the link to the electronic supplementary material.

Supplementary Material 1

Supplementary Material 2

## Data Availability

Data will be made available to bona fide researchers.
