# Supplementary material for: The longitudinal impact of low-dose morphine on diurnal cortisol profiles in people with chronic breathlessness and chronic obstructive pulmonary disease (COPD): an exploratory study
Source: Respir Res. 2025 Apr 23;26:156. doi: 10.1186/s12931-025-03230-9 (PMC12020152; doi:10.1186/s12931-025-03230-9)
Supplement: Supplementary file 1 — Supplementary Material 1 [file 12931_2025_3230_MOESM1_ESM.docx]

Web appendix 1

**Compliance with collection times by study stage**

|  |  | **n**  **(expected number of samples = n x 6)** | **Collection compliance and quality**  **n (row %)** | | | |
| --- | --- | --- | --- | --- | --- | --- |
|  |  |  | **Within 1 hour of designated time** | **Within 3 hours of designated time** | **No time recorded**** | **Missing sample** |
| **Study stage** | **Baseline** | **20 (120)*** | **83 (69.2)** | **4 (3.3)** | **33 (27.5)** | **-** |
|  | **Stage 1**  End of week 1 | **17 (102)** | **55 (53.9)** | **3 (2.9)** | **39 (38.2)** | **5 (4.9)** |
|  | **Stage 3**  End of week 3 | **11 (66)** | **63 (95.5)** | **3 (4.5)** | **-** | **-** |
|  | **Stage 4**  End of 3 months | **7 (42)** | **35 (83.3)** | **1 (2.4)** | **6 (14.3)** | **-** |

*placebo n=6; sustained release morphine 8mg n=8; sustained release morphine 16mg n=6

** There was no statistical difference between compliant and non-compliant sub-groups at any study stage for diurnal cortisol slope nor AUCg.
