# Supplementary material for: The longitudinal impact of low-dose morphine on diurnal cortisol profiles in people with chronic breathlessness and chronic obstructive pulmonary disease (COPD): an exploratory study
Source: Respir Res. 2025 Apr 23;26:156. doi: 10.1186/s12931-025-03230-9 (PMC12020152; doi:10.1186/s12931-025-03230-9)
Supplement: Supplementary file 2 — Supplementary Material 2 [file 12931_2025_3230_MOESM2_ESM.docx]

**The longitudinal impact of low-dose morphine on diurnal cortisol profiles in people with chronic breathlessness and chronic obstructive pulmonary disease (COPD): an exploratory study**

Diana H. Ferreira ^1^

Richella Ryan^2^

Nina Smyth^3^

Angela Clow^3^

David C. Currow^4^

^1^Graduate School of Medicine, Faculty of Science, Medicine and Health, University of Wollongong, Wollongong, New South Wales, Australia.

^2^Arthur Rank Hospice Charity, Cherry Hinton Road, Shelford Bottom, Cambridge, CB22 3FB

^3^ University of Westminster, Regent St, London. W1B 2HW

^4^Faculty of Health, University of Technology Sydney, Sydney, New South Wales, Australia. 2007

**Corresponding author:**

Professor David Currow

Faculty of Health

University of Technology Sydney

Sydney NSW 2007

Email: david.currow@uts.edu.au

**Word count:** 3531

**Tables and figures:** 6

**References** 49

**Key words**: Persistent breathlessness, sustained-release morphine, cortisol, palliative care, symptom control.

**Trial Registration:** Registration Number NCT02720822- date registered 28/03/2016.

**Ethics Declaration**: This study was approved by the Hunter New England Human Research Ethics Committee (Reference 15/12/16/3.06) in accordance with the Declaration of Helsinki.

**Abstract**

**Introduction**

Stress activates the hypothalamic-pituitary-adrenal (HPA) axis of which cortisol is an end product. ‘Allostatic load’ is where systems including the HPA axis are exposed to high, cumulative, physiologic burdens (such as chronic breathlessness) leading to flatter diurnal cortisol slopes and poorer health outcomes.

The aim of this hypothesis-generating study explored longitudinal changes in cortisol secretion and any associated changes in breathlessness after introducing regular, low dose morphine or placebo.

**Methods**

This was an optional, hypothesis-generating sub-study embedded in a multi-site, randomised, double-blind, placebo-controlled trial (RCT) of regular, low-dose morphine for chronic breathlessness and chronic obstructive pulmonary disease. In a blinded dose-increment algorithm by week three, doses were 0mg-32mg. Participants in the RCT could elect to continue in a six-month blinded extension.

This sub-study excluded people who used non-inhaled corticosteroids in the previous month or were on subcutaneous insulin.

Participants collected saliva for cortisol assays for two days: baseline, and ends of weeks 1, 3 and 12 at 3,6 and 12 hours after waking, generating sufficient data to calculate diurnal cortisol slopes and areas under the curve (AUC). Samples were analysed using ELISA.

Correlations between diurnal cortisol profiles (slope and AUC) and a range of measures were explored.

**Results**

Twenty mostly female former smokers were in this sub-study.

At baseline and the end of week 1, one-way ANOVA between-group analyses showed no significant differences in the log-transformed cortisol slope or ln-AUC. There was a strong correlation between the age-adjusted Charlson Comorbidity Index (CCI) and ln-AUC (*r*=-0.70, p<0.001) and moderate correlation with age (*r*=-0.43, p=0.06).

In the blinded extension study, there was a self-selecting blinded group (n=7) all on active medication. Global impression of change (GIC) was highly correlated with the diurnal cortisol slope (rs=0.98, p=0.01), and with *average breathlessness* (r=0.89, p=0.04).

**Discussion**

This hypothesis-generating study did not show a relationship between the diurnal cortisol profile and morphine in people with chronic breathlessness and COPD. For the sub-group still on study at 12weeks, the cortisol curves became steeper as *average breathlessness* decreased and as global impression of change (GIC) improved, suggesting that reducing breathlessness may potentially positively impact the HPA axis in a sub-group of people.

**Key words**: chronic breathlessness, sustained-release morphine, cortisol, palliative care, symptom control.

**Introduction**

Stress is defined as “a state of real or perceived threat to homeostasis that may challenge an organism’s well-being” [1]. The *stress response* is characterised by a complex chain of physiologic and behavioural changes supporting the return to homeostasis [2-4]. This includes activation of the hypothalamic-pituitary-adrenal (HPA) axis which plays a key role in sustaining, modulating and terminating the stress response [1]. Cortisol is an end product of the HPA axis, which is released by the adrenal cortex and responsible for promoting a range of metabolic, cardiovascular, behavioural and immune adaptive responses [5,6]. Cortisol release is regulated by a negative feedback loop to the hypothalamus and pituitary.

‘Allostatic load’ is where allostatic systems such as the HPA axis are exposed to sufficiently high, cumulative, physiologic burden that they become disrupted and damage the body [7,8]. For example, excess production of inflammatory cytokines or chronic stress may disrupt the HPA axis, cardiovascular, and central nervous systems, forcing each system to compensate [9,10]. Higher allostatic loads, identified by at least three different biological markers, were shown to be associated with worse physical and mental health across different settings [8,11,12]. Allostatic load on the HPA axis is classically identified by disruption to the normal patterns of diurnal cortisol secretion [13] and are indicative of disrupted circadian signalling [14]). In particular, flatter diurnal cortisol slopes are associated with a wide range of poorer emotional and physical health [15].

Cortisol can be accurately, conveniently and non-invasively measured in saliva, even in people with a dry mouth [16,17]. In saliva, cortisol is present in its free form and is therefore less prone to fluctuations due to changes in erythrocytes or serum protein – changes that may be present in people with chronic breathlessness.

Changes in the cortisol circadian rhythm may be particularly relevant in chronic unremitting conditions [18]. Importantly, the diurnal cortisol secretion rhythm seems to involve two different patterns [19]: the post-awakening rise in cortisol levels and a basal diurnal cortisol curve reflecting changes from mid-morning to evening. These responses need to be evaluated separately [19].

Chronic breathlessness may be associated with stress and higher allostatic load because it is:

1) a potentially life-threatening sensation;

2) a source of fear and anxiety; and

3) frequently prolonged and progressive, leading high to physiologic burden.

Additionally, people with chronic breathlessness are typically older and have multiple comorbid conditions generating multiple symptoms, contributing to even higher allostatic loads [20,21]. Theoretically, higher allostatic loads may be one of the mechanisms by which chronic breathlessness contributes to premature mortality [22,23].

A link between chronic breathlessness and HPA dysregulation was suggested by one cross-sectional study of 110 participants, comparing the salivary diurnal cortisol profile of people with chronic breathlessness with those of healthy participants [23]. This study revealed that people with moderate-to-severe *breathlessness limiting exertion* (modified Medical Research Council [mMRC] score of 2-4 on a five point ordinal scale) have dysregulation of their normal circadian rhythm of cortisol production generating flatter diurnal cortisol slopes compared with people with mild or no *breathlessness limiting exertion* (mMRC 0 or 1), or healthy controls (p<0.001 in the ANCOVA model for both comparisons).

There is a need to understand better the patterns of cortisol secretion in people with chronic breathlessness. Understanding these patterns is critical to identifying factors that may generate or sustain the observed poorer wellbeing and long-term health outcomes for people who report chronic breathlessness. Further, if the intensity in breathlessness can be reduced, could this be reflected in improved physiological parameters such as restoration of a more normal pattern of diurnal cortisol secretion reflecting a physiological response to better symptom control. Regular, low dose, sustained-release morphine is a potential therapy to reduce the symptoms of chronic breathlessness which requires evaluation [24] of any effect on the HPA axis function.

The aim of this hypothesis-generating study was to explore:

(1) patterns of cortisol secretion in people with chronic breathlessness (extending the earlier cross-sectional findings); and

(2) any changes in patterns of cortisol secretion associated with changes in breathlessness intensity after the introduction of regular, low dose, sustained-release morphine (by dose) or placebo for chronic breathlessness.

**Methods**

This prospective study was an optional, hypothesis-generating sub-study embedded in a multi-site, randomised, double-blind, placebo-controlled trial (RCT) of regular, low-dose, sustained-release morphine for chronic breathlessness associated with chronic obstructive pulmonary disease (COPD). Given block randomisation, there was a representative sample of the varying doses of sustained-release morphine to which people were exposed.

Design and conduct of the RCT are described in detail elsewhere [25]. In brief, participants were randomised to one of three arms: once-daily placebo, sustained-release morphine 8mg or 16mg for one week. Irrespective of the symptomatic response, they were further randomised at weeks 2 and 3 to add placebo or 8mg of sustained release morphine to their initial dose. At week three, doses ranged from 0mg to 32mg of sustained release morphine daily. An optional six-month blinded extension was available to all participants.

In addition to the eligibility criteria for the main study (Web appendix 1), this study required exclusion of people who had oral or intravenous corticosteroids in the previous four weeks (given their disruption of the HPA axis *and* cross-reactivity with salivary cortisol immunoassays; [26]) or were on subcutaneous insulin for diabetes (given described changes in the cortisol awakening response and the diurnal cortisol slope [27,28]).

Participants were asked to collect saliva samples at four timepoints across the whole study (Baseline (day -1, 0), end of week 1 (days 5 and 6; steady state for week 1 dose [29]), end of week 3 (days 19 and 20; steady state for week 3 dose), and blinded extension (after taking stable (blinded) medication for at least three months; Figure 1). For each timepoint, participants were provided with a saliva self-collection pack, a diary for collection days, and an instruction sheet. Each saliva self-collection pack included six saliva-collection devices to collect three daily saliva samples (3, 6 and 12 hours after waking) across two consecutive days generating sufficient data to calculate the diurnal cortisol slope and AUC for each participant at each of the collection timepoints [30,31]. Participants were asked to take steps to minimise stress, especially in the thirty minutes before each sample’s collection.

Saliva samples were collected using the Salivette® Cortisol devices (SARSTEDT, Australia), each consisting of a synthetic swab stored in a plastic tube, together with contemporaneous diary data. Participants were instructed to chew on the swab for one minute before re-storing it in the pre-labelled, colour-coded plastic tube, according to the manufacturers’ instructions. Salivary cortisol is relatively stable at room temperature and did not require freezing but samples were stored in people’s refrigerators until collection by study staff (up to one week later). Samples were frozen at -30^0^ in a central repository until the completion of the whole study and then transported on dry ice to the Stratech^R^ facility in NSW for analysis in September, 2019. All samples underwent one freeze-thaw cycle. On the day of the assay, all samples were thawed and analysed using an Enzyme Linked Immunosorbent Assay (ELISA) developed by Salimetrics LLC (USA), according to the manufacturers’ instructions. Performance testing by Salimetrics indicates a high correlation between salivary cortisol and matched serum cortisol concentrations (r = 0.91). The lower limit of detection (assay sensitivity) is 0.08 nmol/L.

*Sample size and statistical analyses*

Considering that this was a hypothesis-generating study embedded in a larger RCT, no formal power calculation was undertaken. Changes in diurnal cortisol slope and AUC were examined while accounting for other variables which could potentially induce HPA changes, including:

- breathlessness scores measured with a numerical rating scale (NRS) for *worst*, *average*, and *unpleasantness* of breathlessness in the previous 24 hours; higher scores indicate more intense breathlessness [32,33];
- modified Medical Research Council (mMRC) breathlessness scale score; higher scores indicate greater limitations to exertion because of breathlessness [34,35];
- performance status assessed with the Australia-modified Karnofsky Performance Scale (AKPS); lower scores reflect poorer function [36];
- quality of life measured using the EuroQual (EQ-5D-5L) tool [37];
- subjective sleep quality measured with a 4-point Likert scale - from 1 (very good) to 4 (no sleep at all) [38];
- anxiety and depression scores measured with the Hospital Anxiety and Depression Scale (HADS); higher scores indicate the greater likelihood of anxiety and/or depression [39,40];
- global impression of change (GIC) with the study medication (i.e., regular, low-dose, sustained-release oral morphine or placebo) measured with a 7-point Likert scale; from much worse through a neutral perception to much better [41]; and
- harms commonly associated with morphine (constipation, nausea, drowsiness) measured with 4-point Likert scales (higher scores indicate greater harms).

Normality tests (Shapiro-Wilk) were conducted for all numerical variables to determine the appropriate statistical tests. Raw salivary cortisol concentrations were highly skewed and therefore logarithmic transformation (Ln) was employed to normalise data distribution and facilitate inferential statistics [42,43]. The impact of non-compliance in the diurnal cortisol slope and AUC was assessed by comparing means of compliant and non-compliant groups, at each sub-study stage, using unpaired t-tests (with participants with unrecorded collection times included in the non-compliant group).

For Baseline and Week 1, between-arm comparisons were conducted using one-way ANOVA. As the number of participants at Weeks 3 and 4 were smaller, statistical between-arm comparisons were not performed and these data are only described.

Relationships between diurnal cortisol profile (slope and AUCg) and breathlessness measures, other self-assessment measures and harms were explored at each week using Pearson’s or Spearman’s correlation depending on the nature of the variable examined (i.e., numeric or ordinal) and its distribution (i.e., presence or absence of normal distribution). Potential influences of participants’ baseline characteristics on the diurnal cortisol profile (slope and AUCg) were explored using Pearson’s correlation for numerical variables, and unpaired t-tests for comparison of sub-groups defined by categorical variables.

*Ethics approval / additional written informed consent.*

This study was approved as a sub-study of the BEAMS trial [25] by the Hunter New England Human Research Ethics Committee (Reference 15/12/16/3.06) and by the local Research Governance Office at each site in accordance with the Declaration of Helsinki. The BEAMS Trial is registered with the National Institutes of Health Clinical Trials Registration site (NCT02720822). In addition to written, informed consent for the main RCT, participants in this sub-study provided additional written, informed consent.

*Reporting framework and data*

This sub-study is reported using the CONSORT framework for reporting randomised controlled studies. No data were imputed.

**Results**

The main BEAMS study did not show any differences in the reduction of breathlessness between morphine and placebo [25], so differences in breathlessness between groups were not expected in this sub-study. The study explores any impact of regular, low-dose, sustained release morphine on the HPA axis independent of any effects on breathlessness.

*Study sample:*

From the 156 participants included in the BEAMS study, 105 were screened for this sub-study between August 2017 and August 2019: 27 were excluded because they were taking or had taken systemic glucocorticoids in the previous four weeks; 2 were taking insulin for diabetes and 53 chose not to participate. Twenty-three participants were eligible and were included in the cortisol sub-study. Of these people, three were excluded from the analysis for: skipping baseline collections by mistake; collecting invalid baseline samples; and lack of compliance by omission of multiple collection times.

Participants were mostly elderly women who were overweight, able to care for themselves but unable to carry on normal activity or do active work (AKPS ≤ 70; Table 1). Most participants were former smokers and only two participants required long-term oxygen therapy. Approximately one half of the participants had current histories of psychiatric illnesses (e.g., anxiety and/or depression), and more than one half of those people were taking long-term antidepressants. Most participants were medicated with a steroid inhaler, whose doses had been stable for ≥1 week prior to sub-study enrolment.

*Compliance with cortisol sampling:*

Eighty-three (83/120; 69.2%) baseline collections (20 participants times six samples) were collected within one hour of the recommended collection times (Table 2).

*Relationship between cortisol profile and morphine:*

At baseline, the one-way ANOVA between-group analyses showed no significant differences between arms in the Ln-transformed summary measures of diurnal cortisol slope [F (0.24), p=0.79] and AUCg [F (0.06), p=0.94] (Table 3).

In Week 1, there were no significant differences between the three arms (placebo, 8mg morphine daily, 16mg morphine daily) for log-transformed cortisol slope nor for area under the curve (Table 4). There were no correlations for clinically relevant relationships between the diurnal cortisol slope (Table 5).

In Week 3, Ln-transformed mean diurnal cortisol slopes were similar for all three arms: sustained-release morphine ≥16mg (mean ± SD = -0.12 ± 0.06, n=6); sustained-release morphine 8 mg (mean ± SD = -0.11 ± 0.09, n=4); and placebo (-0.10). Ln-AUCg were of a similar order of magnitude and direction: placebo (3.50); sustained-release morphine 16mg (mean ± SD = 3.35 ± 0.92, n=6); and sustained-release morphine 8mg (mean ± SD = 3.05 ± 0.21, n=4; Table 4).

At Week 12, there is a small, self-selecting group (n=7) who continued with the study because they perceived benefit from the intervention while blinded. All were on active medication. Within this group the global impression of change (GIC) and correlation with the diurnal slope was extremely high (rs=0.98, p=0.01). This is in contrast to the findings at the end of Weeks 1 and 3 where no group showed any significant difference.

In the group still on medications at week 12, there were positive correlations between diurnal cortisol slope and *average breathlessness* and *worst breathlessness*, with the slope becoming flatter when both measures were more intense (r=0.89, p=0.04; r=0.79, p=0.11, respectively).

*Relationship between cortisol profile and other clinical measures:*

At baseline, there were no statistically significant correlations between summary cortisol measures (slope and AUCg) and any of the breathlessness measures at Baseline (Table 5). Measures of function and symptom burden that were correlated with the diurnal cortisol slope included: AKPS with the slope flattening as functional status declined (*rs*=-0.66, p=0.001); and sleep quality with the slope flattening as self-reported sleep worsened (*rs*=-0.61, p=0.005). Factors with the potential to influence HPA axis activity and cortisol levels were examined to explore their influence at baseline. There was a strong correlation between the age-adjusted Charlson Comorbidity Index (CCI) and the ln-AUCg, with the ln-transformed AUCg becoming lower for higher comorbidity indices (*r*=-0.70, p<0.001). There was also a moderate correlation between age and Ln-AUCg, with the ln-transformed AUCg becoming lower with more advanced age (*r*=-0.43, p=0.06).

**Discussion**

This hypothesis-generating study did not show a clear relationship between the diurnal cortisol profile and therapy with regular, low-dose, sustained-release morphine in the context of chronic breathlessness associated with COPD. These results are consistent with the main trial, where there were no differences in breathlessness scores between the morphine and placebo groups after one week [25]. Data at baseline provided support for links between diurnal cortisol profiles with age and number of co-morbidities. Worsening physical functioning and poorer sleep were also associated with flatter cortisol curves at baseline. For the sub-group remaining on this study for 3 months, the cortisol curves became steeper as *average breathlessness* decreased and as global impression of change (GIC) improved, suggesting that morphine may potentially have an impact on the HPA axis long term, at least in a sub-group of people.

The study builds directly on the seminal work of Ryan *et al*. [23], replicating the success of monitoring HPA axis function using self-collected saliva sampling in a population of people with COPD and a range of co-morbidities. Determination of cortisol profiles requires saliva sampling according to a strict time schedule, relative to awakening time. [19] Here we chose to collect samples at 3,6 and 12 hours post awakening. The majority of participants collected samples within an hour of the required time. There were no statistically significant differences in cortisol values between samples collected late or on time as all samples were collected after the initial post-awakening burst in cortisol secretion when values are critically time dependent. [19]

The cross-sectional study of Ryan *et al*. [23] demonstrated flatter mean diurnal cortisol slope in people with more intense *breathlessness limiting exertion* (MRC), but not intensity rating of breathlessness potentially reflecting that it is overall function that has the strongest association with changes in diurnal cortisol secretion patterns. The current study would support the direction of these associations at Weeks 1 and 3.

No healthy control group was studied in the current study but it is interesting to cautiously observe values between studies. Ryan *et al*. reported cortisol values at 3-, 6- and 12-hours post awakening for a similar age group of healthy participants as: 15.3±8.2; 8.3±4.1; 2.6±1.4nmol/l. Concentrations at 3 and 6 hours post awakening are clearly higher in that healthy control sample compared to our study sample (and the clinical sample reported in Ryan *et al*.). However, the 12-hour values are similar in both studies for all participants, accounting for the reduced diurnal decline reported. A reduced diurnal decline in cortisol secretion has been associated with a wide range of physical and mental ill-health [15] and is indicative of disrupted circadian function. [14]

In this current study, associations with diurnal cortisol secretion were most evident at baseline, where statistical power was greatest. Although no direct correlations with breathlessness measures were found, data were consistent with expectations with flattened profiles being associated with worse functional status and worse sleep quality. The negative correlation between age and the age-adjusted Charlson Comorbidity Index (CCI) with total cortisol secreted across the day reflects that healthy ageing is associated with greater cortisol secretion [44,45]. We attribute this to circadian dysfunction in this population and the reduced diurnal slope, resulting in lower cortisol concentrations at 3 and 6 hours post awakening, as discussed above.

At three months, the results of a steeper cortisol diurnal slope being associated with more positive global impression of change and less NRS-rated breathlessness are consistent with reports that sub-groups of people respond to morphine administration[24]. These responders may undergo restoration of a more dynamic and healthy pattern of diurnal cortisol secretion [15] and provide the first evidence that symptomatic relief of chronic breathlessness has benefits for broader aspects of wellbeing.

In seeking the first longitudinal pilot data, this current study adds an important dimension to the potential negative impacts of chronic breathlessness (loss of the diurnal pattern of cortisol secretion) and the theoretical benefit of working to relieve the symptom with the potential of a concomitant physiological improvement with systemic benefits. The work opens a wide-ranging line of inquiry into the negative pathophysiological impacts of chronic breathlessness and the potential of reversing some of these impacts with better symptom control (when the underlying reversible cause(s) is/are optimally treated).

A strength of this study is that it is a prospective collection of data when people were still blinded to the arm to which they were randomised allowing analyses to include the subjective sensation of breathlessness in the most objective way. The assay used is a well validated tool and participant compliance was relatively high. Sufficient sampling across 12 hours was considered sufficient to calculate parameters including diurnal decline and AUCg. The study demonstrates the feasibility and need to extend this line of investigation to clearly establish treatment effects upon the HPA axis function.

Limitations include the small sample size that diminished further as time on study progressed and the absence of other supporting physiological measures that may reflect the net impact of reduced symptom burden. Further, only one person had the most severe level of breathlessness (mMRC 4) in this sub-study, with evidence from this trial and earlier studies that people with the most severe chronic breathlessness derive the most symptomatic benefits from regular, low-dose morphine [46].

The study reflects the complexity of changes in patterns of cortisol secretion in people with COPD and chronic breathlessness. It points to the need for larger, prospective studies in people who perceive that their chronic breathlessness has responded symptomatically to the therapy in order to understand more completely any physiological changes that may occur as the pharmacodynamic effects of regular, low dose sustained release morphine are realised.

*Implications for future research*

This current small study is one further step towards understanding any interplay between chronic breathlessness and the HPA axis. Considering the longitudinal changes seen, this study needs to be replicated on a much larger scale in an unblinded consecutive cohort of people commenced on regular, low dose sustained release morphine for the symptomatic reduction of chronic breathlessness. In designing such a study, learning from the challenges of recruitment to the current study will be invaluable. This current study shows that such a study is necessary given that one third of participants, all of whom were on active medication, continued to take that medication at three months and a strong correlation with their diurnal cortisol secretion curves. If there are a cohort of responders who re-establish a ‘healthier’ diurnal pattern of cortisol secretion, does this benefit other measures of health and wellbeing?

*Implications for clinical care*

There are no direct implications for day-to-day clinical care, however there is the possibility that, as clinicians, we have under-estimated the benefits of symptom control, dismissing it in our minds simply as a ‘nice to have’ outcome. Were there measurable systemic benefits, it fundamentally shifts the responsibility of clinicians to be much more proactive in seeking the presence and impact of symptoms, and responding in timely and fulsome ways [47,48].

Most importantly, this work may help to characterise the sub-group of people who can safely benefit from regular, low-dose sustained release morphine for the symptomatic reduction of chronic breathlessness. Although the size of such a group may be relatively small, the benefit that they derive is potentially lifechanging [49].

**Declarations**

**Ethics approval and consent to participate**

Ethics approval was obtained from relevant Health Human Research Ethics Committees before recruitment commenced. All participants gave informed written consent.

**Consent to publish declaration:** not applicable

Thanks go to every participant in this study at a very difficult time of their life. The authors thank Ms. Debbie Marriott for her ready assistance and for her expertise in article formatting and submission.

**Competing Interests Statement:**

The authors DF, RR, NS, AC report no competing interests. DC is an unpaid advisory board member for Helsinn Pharmaceuticals. He is a paid consultant and receives payment for intellectual property with Mayne Pharma and is a consultant with Specialised Therapeutics Australia Pty. Ltd.

**Funding Statement**

This work was supported by the National Health and Medical Research Council, Australia (Grant Number APP1065571) and sponsored by Flinders University, Adelaide, Australia. The funders and study sponsors had no role in the study design and will have no role in the data collection, analysis or dissemination of study results.

**Authors’ Contributions:**

DF, RR, NS, AC and DCC were involved in conceiving and designing the study. DF and DCC were responsible for data analysis. DF and DCC wrote the first draft of the manuscript. All authors contributed to subsequent drafts and were involved in the critical revision of the article for important intellectual content. All authors approved the final version of the article to be published.

**References**

1. Smith SM, Vale WW. The role of the hypothalamic-pituitary-adrenal axis in neuroendocrine responses to stress. Dialogues Clin Neurosci. 2006;8(4):383-95.

2. Chrousos GP, Gold PW. The concepts of stress and stress system disorders. Overview of physical and behavioral homeostasis. JAMA. 1992;267(9):1244-52.

3. De Kloet ER, Joëls M, Holsboer F. Stress and the brain: from adaptation to disease. Nat Rev Neurosci. 2005;6(6):463-475.

4. Ulrich-Lai YM, Herman JP. Neural regulation of endocrine and autonomic stress responses. Nat Rev Neurosci. 2009;10(6):397-409.

5. Charmandari E, Tsigos C, Chrousos G. Endocrinology of the stress response. Annu Rev Physiol. 2005;67:259-84.

6. Sapolsky RM, Romero LM, Munck AU. How do glucocorticoids influence stress responses? Integrating permissive, suppressive, stimulatory, and preparative actions. Endocr Rev. 2000;21(1):55-89.

7. McEwen BS. Stress, adaptation, and disease: Allostasis and allostatic load. Ann N Y Acad Sci. 1998;840:33-44.

8. Seeman TE, McEwen BS, Rowe JW, Singer BH. Allostatic load as a marker of cumulative biological risk: MacArthur studies of successful aging. Proc Natl Acad Sci U S A. 2001;98(8):4770-75.

9. McEwen BS. What is the confusion with cortisol? Chronic Stress. 2019;3:2470547019833647.

10. Schulkin J. Allostasis, homeostasis, and the costs of physiological adaptation. Cambridge University Press; 2004.

11. Guidi J, Lucente M, Sonino N, Fava GA. Allostatic Load and Its Impact on Health: A Systematic Review. Psychother Psychosom. 2021;90(1):11-27.

12. Seeman TE, Singer BH, Rowe JW, Horwitz R, Mcewen B. Price of adaptation—allostatic load and its health consequences: MacArthur studies of successful aging. Arch Intern Med. 1997;157(19):2259-68.

13. Fries E, Hesse J, Hellhammer J, Hellhammer DH. A new view on hypocortisolism. Psychoneuroendocrinology. 2005;30(10):1010-16.

14. Nader N, Chrousos GP, Kino K. Interactions of the circadian CLOCK system and the HPA axis. Trends Endocrinol Metab 2010;21(5):277-86.

15. Adam EK, Quinn ME, Tavernier R, McQuillan MT, Dahlke KA, Gilbert KE. Diurnal cortisol slopes and mental and physical health outcomes: A systematic review and meta-analysis. Psychoneuroendocrinology 2017;83:25–41.

16. Spencer RL, Deak T. A users guide to HPA axis research. Physiol Behav. 2017;178:43-65.

17. Kirschbaum C, Hellhammer DH. Salivary cortisol in psychoneuroendocrine research: recent developments and applications. Psychoneuroendocrinology. 1994;19(4):313-333.

18. Lupien SJ, McEwen BS, Gunnar MR, Heim C. Effects of stress throughout the lifespan on the brain, behaviour and cognition. Nat Rev Neurosci. 2009;10(6):434-45.

19. Smyth N, Clow A. Salivary Bioscience, Human Stress and the Hypothalamic–Pituitary–Adrenal Axis. Salivary Bioscience: Springer, Cham; 2020. p. 89-114.

20. Naughton C, Bennett K, Feely J. Prevalence of chronic disease in the elderly based on a national pharmacy claims database. Age Ageing. 2006;35(6):633-6.

21. Milton JC, Hill-Smith I, Jackson SH. Prescribing for older people. BMJ. 2008;336(7644):606-9.

22. Nishimura K, Izumi T, Tsukino M, Oga T, on Behalf of the Kansai COPD Registry and Research Group in Japan. Dyspnea is a better predictor of 5-year survival than airway obstruction in patients with COPD. Chest. 2002;121(5):1434-40.

23. Ryan R, Clow A, Spathis A, Smyth N, Barclay S, Fallon M, Booth S. Salivary diurnal cortisol profiles in patients suffering from chronic breathlessness receiving supportive and palliative care services: A cross-sectional study. Psychoneuroendocrinology. 2017;79:134-45.

24. Rajnoveanu RM, Harangus A, Todea DA, Man MA, Budin CE, Rajnoveanu AG.

Opioids in Treatment of Refractory Dyspnea in Chronic Obstructive Pulmonary Disease: Yes, No or Maybe. J Pers Med. 2024;14(3):318.

25. Ekström M, Ferreira D, Chang S, Louw S, Johnson MJ, Eckert DJ, et al. Effect of Regular, Low-Dose, Extended-release Morphine on Chronic Breathlessness in Chronic Obstructive Pulmonary Disease: The BEAMS Randomized Clinical Trial. JAMA. 2022;328(20):2022-32.

26. Granger DA, Hibel LC, Fortunato CK, Kapelewski CH. Medication effects on salivary cortisol: tactics and strategy to minimize impact in behavioral and developmental science. Psychoneuroendocrinology. 2009;34(10):1437-48.

27. Joseph JJ, Wang X, Spanakis E, Seeman T, Wand G, Needham B, t al. Diurnal salivary cortisol, glycemia and insulin resistance: The multi-ethnic study of atherosclerosis. Psychoneuroendocrinology. 2015;62:327-35.

28. Joseph JJ, Golden SH. Cortisol dysregulation: the bidirectional link between stress, depression, and type 2 diabetes mellitus. Ann N Y Acad Sci. 2017;1391(1):20-34.

29. Gourlay GK, Plummer JL, Cherry DA. Chronopharmacokinetic variability in plasma morphine concentrations following oral doses of morphine solution. Pain. 1995;61(3):375-81.

30. Adam EK, Kumari M. Assessing salivary cortisol in large-scale, epidemiological research. Psychoneuroendocrinology. 2009;34(10):1423-36.

31. Kraemer HC, Giese-Davis J, Yutsis M, O’Hara R, Neri E, Gallagher-Thompson D, et al. Design decisions to optimize reliability of daytime cortisol slopes in an older population. Am J Geriatr Psychiatry. 2006;14(4):325-33.

32. Gift AG, Narsavage G. Validity of the numeric rating scale as a measure of dyspnea. Am J Crit Care. 1998;7(3):200-204.

33. Wilcock A, Crosby V, Clarke D, Tattersfield A. Repeatability of breathlessness measurements in cancer patients. Thorax. 1999;54(4):374-74.

34. Bestall J, Paul E, Garrod R, Garnham R, Jones PW, Wedzicha JA. Usefulness of the Medical Research Council (MRC) dyspnoea scale as a measure of disability in patients with chronic obstructive pulmonary disease. Thorax. 1999;54(7):581-86.

35. Stenton C. The MRC breathlessness scale. Occup Med (Lond). 2008;58(3):226-7.

36. Abernethy AP, Shelby-James T, Fazekas BS, Woods D, Currow DC. The Australia-modified Karnofsky Performance Status (AKPS) scale: a revised scale for contemporary palliative care clinical practice [ISRCTN81117481]. BMC Palliat Care. 2005;4(1):7.

37. Herdman M, Gudex C, Lloyd A, Janssen MF, Kind P, Oarkin D, et al. Development and preliminary testing of the new five-level version of EQ-5D (EQ-5D-5L). Qual Life Res. 2011;20(10):1727-36.

38. Martins RT, Currow DC, Abernethy AP, Johnson MJ, Toson B, Eckert DJ. Effects of low-dose morphine on perceived sleep quality in patients with refractory breathlessness: A hypothesis generating study. Respirology. 2016;21(2):386-91.

39. Hopwood P, Howell A, Maguire P. Screening for psychiatric morbidity in patients with advanced breast cancer: validation of two self-report questionnaires. Br J Cancer. 1991;64(2):353-56.

40. Zigmond AS, Snaith RP. The hospital anxiety and depression scale. Acta psychiatrica scandinavica. 1983;67(6):361-370.

41. Oxberry SG, Bland JM, Clark AL, Cleland JGF, Johnson MJ. Minimally clinically important difference in chronic breathlessness: every little helps. Am Heart J. 2012;164(2):229-35.

42. Smyth JM, Ockenfels MC, Gorin AA, Catley D, Porter LS, Kirschbaum C, et al. Individual differences in the diurnal cycle of cortisol. Psychoneuroendocrinology. 1997;22(2):89-105.

43. Sephton SE, Sapolsky RM, Kraemer HC, Spiegel D. Diurnal cortisol rhythm as a predictor of breast cancer survival. J Natl Cancer Inst. 2000;92(12):994-1000.

44. Menet JS, Rosbash M. When brain clocks lose track of time: cause or consequence of neuropsychiatric disorders. Curr Opin Neurobiol 2011;21(6):849-857.

45. Van Cauter E, Leproult R, Kupfer DJ. Effects of gender and age on the levels and circadian rhythmicity of plasma cortisol. J Clin Endocrinol Metab 1996;81(7):2468-73.

46. Johnson MJ, Bland JM, Oxberry SG, Abernethy AP, Currow DC. Opioids for chronic refractory breathlessness: patient predictors of beneficial response. Eur Resp J 2013;42(3):758-766.

47. Ahmadi Z, Sandberg J, Shannon-Honson A, Vandersman Z, Currow DC, Ekstrom M. Is chronic breathlessness less recognised and treated compared with chronic pain? A case-based randomised controlled trial. Eur Respir J.2018;52(3):1800887.

48. Kochovska S, Chang S, Ferreira D, Brunelli VN, Luckett T, Morgan L, et al. Invisibility of breathlessness in clinical consultations: a cross-sectional, national online survey. Eur Respir J. 2022;60(5):2201603.

49. Ferreira DH, Ekström M, Verberkt C, Janssen DJA, Currow DC. Is being able to walk to the letterbox life-changing? A qualitative assessment of measures of improvement in persistent breathlessness. ERJ Open Res. 2023:9(2):00530-2022.

**Table 1 - Baseline characteristics of the 20 participants of the cortisol sub-study (clinician assessment measures), separated by study arm according to their allocation at baseline**

| **Characteristics** | | **Placebo (n=6)**  mean ± SD  median (IQR) | **SR morphine 8 mg (n=8)**  mean ± SD  median (IQR) | **SR morphine 16 mg (n=6)**  mean ± SD  median (IQR) | **Differences between arms*** |
| --- | --- | --- | --- | --- | --- |
| **Age (years)** | | 71.8 ± 2.9  72.0 (70.0 – 74.3) | 68.3 ± 8.3  69.0 (62.0 – 71.0) | 72.0 ± 12.4  74.0 (65.5 – 80.5) | H(2)=1.69, p=0.26 |
| **Sex (male)** | | 5 (83.3%) | 3 (37.5%) | 1 (16.7%) | χ^2^=5.69, p=0.06 |
| **BMI (kg/m^2^)** | | 30.0 ± 5.3  30.9 (25.2 – 34.5) | 31.9 ± 7.5  33.0 (29.0 – 35.4) | 24.8 ± 8.2  22.7 (17.4 – 33.1) | F=1.66, p=0.22 |
| **Charlson Comorbidity Index - age adjusted** | | 5.2 ± 1.2  4.8 (4.0 – 6.3) | 4.8 ± 1.5  5.0 (4.0 – 6.0) | 5.3 ± 1.0  6.0 (4.0 – 6.0) | H(2)=0.64, p=0.73 |
| **AKPS** | **80** | - | 2 (25.0%) | 2 (33.3%) | H(2)=1.40, p=0.50 |
|  | **70** | 4 (66.7%) | 3 (37.5%) | 3 (50.0%) |  |
|  | **60** | 2 (33.3%) | 3 (37.5%) | 1 (16.7%) |  |
| **Smoking status** | **Current smoker** | - | 2 (25.0%) | 1 (16.7%) | χ^2^=3.56, p=0.47 |
|  | **Former smoker** | 6 (100%) | 5 (62.5%) | 5 (83.3%) |  |
|  | **Never smoked** | - | 1 (12.5%) | - |  |
| **End tidal CO_2_ (mmHg)^a^** | | 30.0 ± 7.3  32.0 (21.8 – 35.3) | 29.4 ± 4.6  28.5 (26.1 – 34.0) | 24.8 ± 4.8  26.3 (21.3 – 28.3) | F=1.63, p=0.23 |
| **Supplemental oxygen** | | 1 (16.7%) | 1 (12.5%) | 1 (16.7%) | χ^2^=2.84, p=0.58 |
| **Relevant health conditions** | Diabetes mellitus  (type I or II) | 0 (0.0%) | 1 (12.5%) | 1 (16.7%) | χ^2^=1.02, p=0.60 |
|  | Current history of psychiatric illness | 3 (50%) | 4 (50%) | 2 (33.3%) | χ^2^=0.47, p=0.79 |
|  | Previous history of psychiatric illness | 0 (0%) | 1 (12.5%) | 0 (0%) | χ^2^=1.93, p=0.38 |
| **Relevant Medication** | Steroid inhaler | 4 (66.7%) | 7 (87.5%) | 7 (100%) | χ^2^=2.68, p=0.26 |
|  | Anti-depressant | 3 (50.0%) | 2 (25.0%) | 1 (16.7%) | χ^2^=1.75, p=0.42 |
|  | Hormone therapy | 0 (0%) | 1 (12.5%) | 0 (0%) | χ^2^=1.58, p=0.45 |
|  | Immunosuppressant | 0 (0%) | 2 (25%) | 1 (16.7%) | χ^2^=1.70, p=0.43 |
|  | Chemo- or radiotherapy | 0 (0%) | 0 (0%) | 0 (0%) | - |
| **Availability of a carer** | | 4 (66.7%) | 6 (75.0%) | 5 (83.3%) | χ^2^=0.07, p=0.97 |
| **mMRC** | Grade 3 | 5 (83.3%) | 8 (100%) | 6 (100%) | χ^2^=2.46, p=0.29 |
|  | Grade 4 | 1 (16.7%) | - | - |  |
| **Breathlessness** | *Worst* | 6.3 ± 2.2  6.0 (4.6 – 8.6) | 4.5 ± 1.5  4.8 (3.0 – 5.9) | 5.9 ± 1.5  6.3 (4.6 – 6.9) | F=2.18, p=0.14 |
|  | *Average* | 5.1 ± 2.3  4.0 (3.4 – 7.8) | 3.7 ± 1.1  3.8 (2.6 – 4.9) | 4.7 ± 1.4  4.8 (3.4 – 5.5) | H(2)=1.67, p=0.43 |
|  | *Distress caused by breathlessness* | 3.8 ± 3.4  2.3 (1.3 – 7.5) | 2.8 ± 2.4  2.3 (0.5 – 4.8) | 4.3 ± 2.9  4.8 (1.3 – 6.3) | F=0.45, p=0.64 |
| **Quality of life (EQ-5D-5L, VAS)** | | 65.7 ± 23.9  60.0 (52.5 – 88.5) | 51.3 ± 18.9  52.5 (32.5 – 71.3) | 62.5 ± 24.8  62.5 (37.5 – 90.0) | F=0.83; p=0.45 |
| **Hospital Anxiety and Depression Scale (HADS)** | HADS (Anxiety, 0-21) | 7.0 ± 4.9  6.0 (3.8 – 12.3) | 7.3 ± 4.2  8.0 (4.0 – 11.0) | 4.8 ± 3.8  5.0 (1.5 – 7.8) | F=0.61; p=0.56 |
|  | HADS (Depression, 0-21) | 6.2 ± 4.1  5.5 (3.3 – 9.3) | 6.3 ± 3.9  5.0 (3.0 – 9.0) | 4.5 ± 3.8  3.0 (1.8 – 8.0) | F=0.41; p=0.67 |
| **Quality of sleep** | Very good | 1 (16.7%) | 1 (12.5%) | 1 (16.7%) | χ^2^=7.95, p=0.24 |
|  | Good | 4 (66.7%) | 3 (37.5%) | 1 (16.7%) |  |
|  | Poor | - | 4 (50%) | 4 (66.7%) |  |
|  | No sleep at all | 1 (16.7%) | - | - |  |
| BMI = body mass index, VAS = Visual Analogue Scale. Symbols: *Statistical tests performed in order of reporting: Kruskal-Wallis test (non-normally distributed numerical variables), Chi-squared test (categorical variables), and one-way ANOVA (normally distributed numerical variables),.  *^a^Measured with a portable capnography device during quiet breathing.* | | | | | |

**Table 2 - Compliance with collection times by study stage**

|  |  | **n**  **(expected number of samples = n x 6)** | **Collection compliance and quality**  **n (row %)** | | | |
| --- | --- | --- | --- | --- | --- | --- |
|  |  |  | **Within 1 hour of designated time** | **Within 3 hours of designated time** | **No time recorded**** | **Missing sample** |
| **Study stage** | **Baseline** | **20 (120)*** | **83 (69.2)** | **4 (3.3)** | **33 (27.5)** | **-** |
|  | **Stage 1**  End of week 1 | **17 (102)** | **55 (53.9)** | **3 (2.9)** | **39 (38.2)** | **5 (4.9)** |
|  | **Stage 3**  End of week 3 | **11 (66)** | **63 (95.5)** | **3 (4.5)** | **-** | **-** |
|  | **Stage 4**  End of 3 months | **7 (42)** | **35 (83.3)** | **1 (2.4)** | **6 (14.3)** | **-** |

*placebo n=6; sustained release morphine 8mg n=8; sustained release morphine 16mg n=6

** There was no statistical difference between compliant and non-compliant sub-groups at any study stage for diurnal cortisol slope nor AUCg.

**Table 3 - Baseline cortisol mean/median values in each arm-**

|  | Placebo  mean ± SD  median (IQR)*  (n=6) | SR Morphine 8mg  mean ± SD  median (IQR)*  (n=8) | SR Morphine 16mg  mean ± SD  median (IQR)*  (n=6) | Between-arm differences^†^ |
| --- | --- | --- | --- | --- |
| 3-hour cortisol  (nmol/L) | 4.99 ± 4.7  4.73 (2.7 – 7.4) | 8.69 ± 5.6  7.93 (4.3 – 13.3) | 6.10 ± 3.3  5.08 (3.4 – 9.5) | - |
| 6-hour cortisol  (nmol/L) | 5.69 ± 4.5  4.23 (2.2 – 8.9) | 4.01 ± 2.1  3.77 (2.3 – 5.7) | 4.62 ± 1.8  4.13 (3.09 – 6.3) | - |
| 12-hour cortisol  (nmol/L) | 2.28 ± 1.2  2.04 (1.1 – 3.5) | 2.90 ± 2.1  2.04 (1.6 – 3.9) | 2.53 ± 1.34  2.23 (1.9 – 2.4) | - |
| Ln diurnal slope^†^  (Ln nmol/L/hr) | -0.09 ± 0.07 | -0.12 ± 0.09 | -0.10 ± 0.07 | F=0.24, p=0.79 |
| Cortisol AUCg  (nmol.hr/L) | 39.9 ± 23.7  29.9 (24.9 – 64.6) | 39.8 ± 13.4  42.9 (28.8 – 49.3) | 37.5 ± 12.6  31.5 (29.1 – 51.5) | - |
| Ln cortisol AUCg^†^  (Ln nmol.hr/L) | 3.51 ± 0.62 | 3.59 ± 0.40 | 3.57 ± 0.32 | F=0.06, p=0.94 |
| *Values are mean ± standard deviation (SD) and median (interquartile range), except for variables with normal distribution for which values are presented as mean ± SD only.  †Analysis conducted only in Ln-transformed cortisol measures with normal distribution (Levene’s test p≥0.05). | | | | |

**Table 4 – End of 1 week of therapy: cortisol mean/median values in each arm**

|  | Placebo  mean ± SD  median (IQR)  (n=6) | SR Morphine 8mg  mean ± SD  median (IQR)  (n=8) | SR Morphine 16mg  mean ± SD  median (IQR)  (n=6) | Between-arm differences^†^ |
| --- | --- | --- | --- | --- |
| 3-hour cortisol  (nmol/L) | 10.35 ± 6.5  9.96 (5.2 – 13.8) | 6.46 ± 4.2  4.87 (3.7 – 7.8) | 5.52 ± 2.0  6.18 (3.0 – 7.1) | - |
| 6-hour cortisol  (nmol/L) | 5.78 ± 4.1  4.18 (3.8 – 6.9) | 3.40 ± 1.8  3.02 (1.9 – 4.5) | 3.67 ± 1.5  3.44 (2.3 – 4.9) | - |
| 12-hour cortisol  (nmol/L) | 3.26 ± 2.1  2.45 (1.9 – 4.7) | 2.49 ± 1.5  2.01 (1.5 – 3.0) | 2.90 ± 1.6  2.54 (1.5 – 4.3) | - |
| Diurnal slope  (Ln nmol/L/hr) | -0.12 ± 0.10 | -0.10 ± 0.05 | -0.08 ± 0.06 | F=0.35, p=0.71 |
| Cortisol AUCg  (nmol.hr/L) | 51.3 ± 22.9  44.0 (34.5 – 75.3) | 34.8 ± 16.4  28.5 (25.0 – 48.6) | 34.4 ± 8.3  35.2 (26.1 – 41.6) | - |
| Ln cortisol AUCg  (Ln nmol.hr/L) | 3.85 ± 0.39 | 3.44 ± 0.45 | 3.49 ± 0.28 | F=1.56, p=0.24 |
| *Values are mean ± standard deviation (SD) and median (interquartile range), except for variables with normal distribution for which values are presented as mean ± SD only.  †Analysis conducted only in Ln-transformed cortisol measures with normal distribution (Levene’s test p≥0.05).  AUCg – area under the curve (ground) | | | | |

Table 5 – Correlations between study measures and diurnal cortisol slope

|  | **Ln- Diurnal slope** | | | |
| --- | --- | --- | --- | --- |
|  | **Baseline**  (n=20) | **End of week 1**  (n=17) | **End of week 3**  (n=11) | **At least 3 months on the blinded extension study**  (n=7) |
| ***Worst***  **(NRS)** | *r*=0.33  p=0.15 | *r*=0.27  p=0.29 | *r*=0.33  p=0.36 | *r*=0.79  p=0.11 |
| ***Average***  **(NRS)** | *rs*=0.30  p=0.21 | *rs*=0.31  p=0.23 | *r*=0.08  p=0.82 | *r*=0.89  p=0.04 |
| ***Distress***  **(NRS)** | *r*=-0.01  p=0.98 | *r*=0.02  p=0.93 | *r*=-0.37  p=0.29 | *r*=0.10  p=0.87 |
| **mMRC** | *rs*=-0.18  p=0.45 | *rs*=-0.45  p=0.07 | *rs*=-0.56  p=0.08 | *-* |
| **AKPS** | *rs*=-0.66  p=0.001 | *rs*=-0.12  p=0.66 | *rs*=-0.24  p=0.49 | *rs*=0.00  p=1.00 |
| **QOL (EQ-5D-5L VAS)** | *r*=0.14  p=0.56 | *r*=-0.14  p=0.59 | *-* | *rs*=-0.62  p=0.27 |
| **Subjective sleep quality** | *rs*=-0.61  p=0.005 | *rs*=-0.42  p=0.10 | *rs*=-0.29  p=0.43 | *rs*=0.58  p=0.31 |
| **HADS – anxiety** | *r*=-0.15  p=0.54 | *r*=0.04  p=0.89 | *-* | *-* |
| **HADS – depression** | *r*=0.09  p=0.73 | *r*=-0.50  p=0.04 | *-* | *-* |
| **Global impression of change** | *-* | *rs*=0.03  p=0.91 | *rs*=0.10  p=0.78 | *rs*=0.98  p=0.01 |

NRS = Numerical rating scale; mMRC = modified Medical Research Council scale; AKPS = Australian Karnofsky Performance Status; QOL (EQ-5D-5L) **=** Quality of life measured using the EQ-5D-5L questionnaire; HADS = Hospital Anxiety and Depression Scale. Subjective sleep quality measured with a 4-point Likert scale; Global impression of change measured with a 7-point Likert scale. Statistically significant correlations indicated in light grey.
